# Risk factors for adverse events induced by immune checkpoint inhibitors in patients with non-small-cell lung cancer: a systematic review and meta-analysis

**DOI:** 10.1007/s00262-021-02996-3

**Published:** 2021-06-30

**Authors:** E. Suazo-Zepeda, M. Bokern, P. C. Vinke, T. J. N. Hiltermann, G. H. de Bock, G. Sidorenkov

**Affiliations:** 1 Department of Epidemiology, Graduate School of Medical Sciences, University of Groningen, Zusterhuis, Hanzeplein 1, Groningen, The Netherlands; 2 Department of Pulmonology, University of Groningen, University Medical Center Groningen, Groningen, Netherlands

**Keywords:** Lung cancer, Immunotherapy, Adverse events, Immune checkpoint inhibitors

## Abstract

**Background:**

Immune checkpoint inhibitors (ICIs) can cause serious immune-related adverse events (irAEs). This study aimed to identify risk factors for all types of irAEs induced by ICIs in patients with non-small-cell lung cancer (NSCLC), by systematic review and meta-analyses.

**Methods:**

A systematic search was performed in Pubmed, Embase and Web of Science by two independent reviewers. Studies were selected that included patients with NSCLC and evaluated characteristics of patients with and without irAEs induced by ICIs. Quality and risk of bias of the selected studies were assessed. Random effects meta-analyses were conducted to estimate pooled odds ratios (ORs) for risk factors of developing all type of irAEs, and separately for pneumonitis, interstitial lung disease and severe irAEs. With the objective of exploring sources of heterogeneity, stratified analyses were performed by quality and region.

**Results:**

25 studies met the inclusion criteria. In total, the data of 6696 patients were pooled. 33 different risk factors for irAEs were reported. irAEs of interest were reported for 1653 (25%) of the patients. Risk factors related to the development of irAEs were: C-reactive protein, neutrophil lymphocyte ratio (NLR), use of PD-1 inhibitor, high PD-L1 expression, an active or former smoking status, ground glass attenuation, and a better treatment response.

**Conclusion:**

The identified risk factors for the development of these irAEs are mostly related to the alteration of the immune system, proinflammatory states and loss of immunological self-tolerance. Patients identified as having a higher risk for irAEs should be monitored more closely.

**Supplementary Information:**

The online version contains supplementary material available at 10.1007/s00262-021-02996-3.

## Introduction

Lung cancer is one of the most commonly diagnosed cancers in the world, as well as the leading cause of cancer death in men and women [1]. It ranks as one of the cancers with the lowest survival, being about 50% within 1 year after diagnosis, and 20% within 5 years [2]. Of all lung cancer cases, more than 85% percent are classified as non-small-cell lung cancer (NSCLC), and treatment efforts have mainly focused on this histological type [3].

In recent years, new treatments have emerged as an effective option for patients diagnosed with advanced NSCLC. Patients with advanced NSCLC especially benefit from the introduction of immunotherapy with immune checkpoint inhibitors (ICIs), which have shown better efficacy and safety compared to older treatments like chemotherapy and radiotherapy [4]. At present, the ICIs that target programmed cell death 1 (PD-1)/programmed cell death-ligand 1 (PD-L1) and the cytotoxic T-lymphocyte-associated protein 4 (CTLA 4), allowing the activation of the T lymphocytes antineoplastic activity, are approved by the Food and Drug Administration (FDA) for the treatment of advanced NSCLC [5, 6]. The ICIs can cause immune-related adverse events (irAEs), the incidence of which, depending on their degree, varies between 24 and 38% [7–12]. These irAEs are due to the alteration of immunological self-tolerance caused by the blockade of the immune checkpoint receptors PD-1/PD-L1 and CTLA-4, and are different from adverse events (AEs) after chemotherapy and radiotherapy [13]. The skin, liver, gastrointestinal, pulmonary and endocrine organs are most commonly affected [14, 15].

The occurrence of severe irAEs requiring immunosuppression and complete cessation of ICI treatment is estimated between 9 and 33% [7–11]. The high cost of these therapies and the severity of the irAEs create a need to identify risk factors for AEs of immunotherapy that can be taken into account when choosing a therapy for patients with NSCLC [16]. Previous studies described AEs induced by ICIs [17, 18], and other studies that evaluated characteristics that predispose patients treated with ICIs to irAEs as well [19–21]. Although previous systematic reviews have evaluated risk factors for adverse events, they either focus on a limited number of risk factors (like tumor or ICI class) [20], or on selected adverse events, such as fatal toxicities [21]. Therefore, we aimed to review and meta-analyze the results of published studies to identify risk factors for irAEs, not limited to specific risk factor domains or types of irAEs. Since irAE patterns do differ per tumor type [20], we restricted this review to studies reporting data on patients with NSCLC.

## Methods

### Review registration

This systematic review was registered in the PROSPERO International prospective register of systematic reviews with the registration number CRD42020194101. The research protocol was prepared following the Guidance notes for registering a systematic review protocol with PROSPERO [22].

### Data sources and researches

Three databases were searched, Pubmed, Embase and Web of Science. Studies published in English between January 1st, 2000 and November 12th, 2020 were reviewed. Key search terms in developing the search strategy were the following: lung cancer, lung neoplasms, immunomodulators, immune checkpoint inhibitors, and specific drug names of ICIs (nivolumab, pembrolizumab, atezolizumab, durvalumab, cemiplimab, avelumab). The search strategy is presented in Supplementary material 1.

### Study selection

Studies were included if the following inclusion criteria were met: (1) study was designed as a randomized clinical trial, cohort or case–control study, (2) patients were diagnosed with NSCLC, and (3) treated with ICIs (4) the study evaluated characteristics of patients presenting adverse events induced by ICIs, specifically PD-1/PDL-1 and CTLA-4 protein blockers. Studies were excluded, (1) if these only evaluated the safety and effectiveness of ICIs and did not evaluate the relationship between risk factors and irAEs; or (2) included less than 10 participants. Three reviewers (ES-Z, MB, GS) performed an initial review and selection of the publications independently according to their title and abstract. Subsequently, two reviewers (ES-Z, MB) read the full text of these publications for their final inclusion. Disagreements in study selection were mediated by a third reviewer (GS).

### Data extraction

The main outcome was the occurrence of any type of immunotherapy-induced adverse events (irAEs), defined as all disorders with a potential immunological background that resulted from the use of ICIs. Included in this definition were a broad variety of chemically induced adverse conditions caused by toxicity, drug interactions, and metabolic events of ICIs.

The Cochrane data collection form [23] (Supplementary material 2) adapted to our study and research question was used independently by the reviewers (MB and ES-Z) to collect data from the selected publications, which was always checked by a third reviewer (PCV). The following information was extracted: first author, year of publication, region, publication type, study funding source, study design, inclusion and exclusion criteria, total number of patients, outcomes (type of irAEs, e.g., ILD or pneumonitis), secondary outcomes (common irAEs, e.g., skin reactions or mucositis), risk factors, methods of recruitment of participants, follow-up time, irAEs reporting rate, and the frequency of each specific grade AE. The odds ratio (OR) for each risk factor was retrieved from the studies. When the OR adjusted for confounders was not available, the crude OR was used or calculated based on the frequency tables (if available). If indicated, ORs and confidence intervals were reversed rising the value to the -1 power (OR^−1^ [Lower CI^−1^; Upper CI^−1^]) to harmonize the reference categories within each risk factor.

### Quality assessment

Two reviewers (MB and ES-Z) assessed the quality and risk of bias of the selected papers using the Cochrane Collaboration’s tool for assessing risk of bias in the case of Randomized clinical trials, or the Quality in Prognostic Studies (QUIPS) [24] in the case of non-randomized studies (case–control studies and cohort studies). The “Scottish Intercollegiate Guidelines Network: rating a quality of Cohort Studies” was used to classify papers into high quality, acceptable quality and low quality. Disagreements in the study selection, data collection, and quality assessment steps were mediated by a third reviewer (GS) and discussed until consensus was reached.

### Data synthesis and analysis

Study characteristics as well as study outcomes were described. When at least two studies reported data on the same risk factor, random effects meta-analysis models were performed including both crude and adjusted ORs to provide pooled ORs and the related 95% confidence intervals (95%CI), where the outcome was the occurrence of any irAEs. A stratified analysis was performed for interstitial lung disease (ILD), pneumonitis, severe irAEs. The included studies classified as severe those irAEs grade 3 or higher according to the Common Terminology Criteria for Adverse Events (CTCAE). This pooling was done under the assumption of homogeneity. To evaluate the justification of this assumption, the *χ*^*2*^ test statistic *I*^*2*^ was calculated. A value of *I*^*2*^ > 50% was considered as heterogeneous. To explore sources of heterogeneity, stratified analyses were performed by quality/risk of bias (high, moderate and low risk of bias), and study region (Asia vs USA/Europe). Sensitivity analyses were performed by excluding the crude ORs to assess the validity of the meta-analysis. Analyses were performed using Stata software, version 14.0 (StataCorp).

## Results

From the three databases, after deleting duplicates, 221 potentially eligible articles were obtained for title and abstract screening after deleting duplicates (Supplementary Fig. 1). After the initial screening, 45 articles were selected for full text reading, of which 25 met the inclusion and exclusion criteria. The reasons for exclusion were: 11 studies did not stratify by type of cancer (NSCLC/ other cancers), one study included less than 10 patients, six studies did not assess risk factors for adverse events, one study did not stratify by type of treatment (chemotherapy/ immunotherapy) and one study included the same group of patients and risk factors twice.

A total of 6696 unique patients were included in all studies. The number of patients included per study ranged from 42 to 1548 (Table 1). 14 studies took place in Japan (1859 patients), seven in the United States of America (USA) (3179 patients), two in China (366 patients) and two in Europe (1292 patients). irAEs of interest were reported for 1653 (25%) of the patients. 33 different risk factors for irAEs were reported by the studies included in the meta-analyses. The most frequently reported risk factors were sex (19 studies), ECOG performance status (16 studies), histology (16 studies), smoking status (13 studies) and age (12 studies) (Supplementary able 1). The included studies focused on the following irAEs: all types of irAEs combined (8 studies), ILD (6 studies), pneumonitis (5 studies), severe irAEs (3 studies), acute exacerbation of ILD (1 study), infectious diseases (1 study), skin reactions (1 study), immune-related thyroid dysfunction (1 study), ICI-related cardiotoxicity (1 study) (see Table 1).

**Table 1:** Study characteristics

Author	Year	Location	Participants	Outcome	Type of risk factor
Ahmed T [45]	2020	USA	285	All type of irAEs	Performance status, age
Aso M [46]	2020	Japan	155	Skin reactions	Histology, PD-L1 expression, performance status, preexisting anti-nuclear antibodies, anti-thyroid antibodies, rheumatoid factor, sex, smoking status
Chu X [27]	2020	China	300	Pneumonitis	Age, sex, smoking status, performance status, treatment line, treatment combination, preexisting lung diseases, use of steroids, eosinophil count
Cortellini A [47]	2020	Italy	1010	All type of irAEs	Sex, age, performance status, histology, number of organs affected
Duma N [34]	2019	USA	231	All type of irAEs, severe irAEs	Sex
Fujita K [26]	2019	Japan	167	Infectious diseases	Age, ALK gene, asthma, bronchiectasis, COPD, corticosteroids before nivolumab, corticosteroids during/after nivolumab, diabetes EGFR mutation, histology, history of thoracic surgery, hypothyroidism, ILD, initial stage no. cycles, performance status, radiotherapy, rheumatoid arthritis, sex, smoking status
Fukihara J [48]	2019	Japan	170	Pneumonitis	Abnormal CT findings, age, albumin, ALT, AST, creatinine CRP, emphysema, histology, ILD, NLR, past thoracic RT, pembrolizumab treatment, performance status, sex, smoking pack-years, treatment line, white blood cell count
Kichenadasse G [49]	2020	USA	1548	All type of irAEs	Sex, ethnicity, performance status, histology, treatment line, smoking status, PD-L1 positive vs negative, LDH, lung immune prognostic index
Koyama J [25]	2019	Japan	132	Immune-related thyroid dysfunction	Age, EGFR/ALK positive, histology PD-L1 expression, performance status, sex, smoking status, thyroid dysfunction, treatment line, TTF1 expression
Metro G [50]	2020	Greece, Italy, Spain, Switzerland	282	All type of irAEs	Brain metastasis
Moey M [51]	2020	USA	196	Inhibitor-related cardiotoxicity	Ethnicity, concomitant irAEs, COPD, diabetes, hyperlipidemia, hypertension, PD-(L)1 agent, previous atrial fibrillation/flutter, previous cardiovascular disease, previous cerebrovascular disease, previous chronic kidney disease, radiotherapy, sex, stage, type of lung tumor (NSCLC), use of alkylating agents, use of anti-topoisomerase drugs, use of anti-VEGF drugs, use of antimetabolites, use of beta-blocker, use of calcium channel blocker, use of loop diuretics, use of renin–angiotensin–aldosterone system inhibitors, use of statins, use of steroids, use of taxanes, use of thiazide diuretic
Nakahama K [30]	2018	Japan	201	ILD	CRP, histology, IAOT, LDH, performance status, pleural effusion, previous pneumonitis pulmonary metastasis, sex, smoking status
Nakanishi Y [29]	2019	Japan	83	ILD	Abnormal CT findings, age CRP, driver mutation, emphysema, ground glass attenuation, histology history of radiation pneumonitis, honeycombing, KL-6 in serum, LDH, lymphocyte count, neutrophil count, NLR, PD-L1 expression, performance status, preexisting ILD, prior thoracic RT, reticular shadow, sex, smoking status, white blood cell count
Nishiyama N [28]	2019	Japan	48	Acute exacerbation of ILD	Age ground glass attenuation, performance status, sex, smoking pack-years, UIP radiological
Okada N [32]	2020	Japan	102	ILD	COPD, histology, ILD, PD-(L)1 agent, PD-L1 expression ≥ 50% vs < 50%, performance status, radiotherapy, sex, smoking pack-years, stage, treatment line, use of EGFR-TKI
Owen DH [52]	2018	USA	91	Pneumonitis	ALI, heavy smoking history, histology, KRAS mutation, NLR non-CNS radiation after ICI, PLR, RT after ICI, sex, smoking status, thoracic/chest wall RT
Sakata Y [53]	2019	Japan	42	Severe irAEs	Age, histology, initial stage PD-L1 expression, pembrolizumab treatment, performance status, sex, smoking status, treatment line, tumor burden
Shankar B [54]	2020	USA	623	All type of irAEs	Histology, non-white ethnicity, PD-(L)1 agent, performance status, sex, smoking status, stage, treatment combination, treatment response
Sugano T [31]	2020	Japan	130	ILD	Age, histology, performance status, previous interstitial pneumonia, radiotherapy, sex, smoking status
Suresh K [55]	2018	USA	205	Pneumonitis	Age, ethnicity, chemotherapy, combination ICI treatment, histology, initial stage, other treatments, pembrolizumab treatment, ethnicity, sex, smoking status, surgery
Suzuki Y [56]	2020	Japan	138	ILD	Asthma, best supportive care after ICIs, COPD, diabetes, digestive ulcer, hepatic disease, histology, hypertension, PD-L1 expression ≥ 50% vs < 50%, performance status, previous cardiovascular disease, previous cerebrovascular disease, sex, smoking status, stage, treatment line, treatment response
Toi Y [57]	2019	Japan	137	All type of irAEs	Histology, performance status, preexisting anti-nuclear, antibodies, anti-thyroid antibodies, rheumatoid factor, sex, smoking status
Watanabe S [58]	2020	Japan	231	ILD	Cryptogenic organizing pneumonia-like, histology, PD-L1 expression ≥ 50% vs < 50%, pembrolizumab treatment, performance status, sex, smoking status, stage, treatment line
Yamaguchi T [33]	2018	Japan	123	Pneumonitis	Age, CRP, emphysema score, fibrosis, histology, LDH performance status, sex, smoking status, treatment line
Zeng X [59]	2020	China	66	All type of irAEs	Comorbidity index

*PD-L1* programmed death-ligand 1; *ALK* anaplastic lymphoma kinase; *COPD* chronic obstructive pulmonary disease; *EGFR* epidermal growth factor receptor; *NSCLC* non-small-cell lung carcinoma; *irAEs* immune-related adverse events; *ILD* interstitial lung disease; *CRP* C-reactive protein; *CT* computed tomography; *ALT* alanine transaminase; *AST* aspartate transaminase; *NLR* neutrophil to lymphocyte ratio; *RT* radiotherapy; *TTF1* transcription termination factor 1; *LDH* lactate dehydrogenase; *ICI* immune checkpoint inhibitors; *UIP* usual interstitial pneumonia; *CNS* central nervous system; *PLR* platelet lymphocyte ratio; *IAOT* imaging findings of airway obstruction adjacent to lung tumors; *VEGF* vascular endothelial growth factor

Six studies were graded as having high quality/low risk of bias, eight as having moderate quality/risk of bias and eleven as having low quality/high risk of bias (Supplementary Fig. 2). Low quality papers had important problems in statistical analysis and reporting problems (9 studies) or did not provide a clear definition of confounders or adjustment (8 studies).

**Fig. 1:**
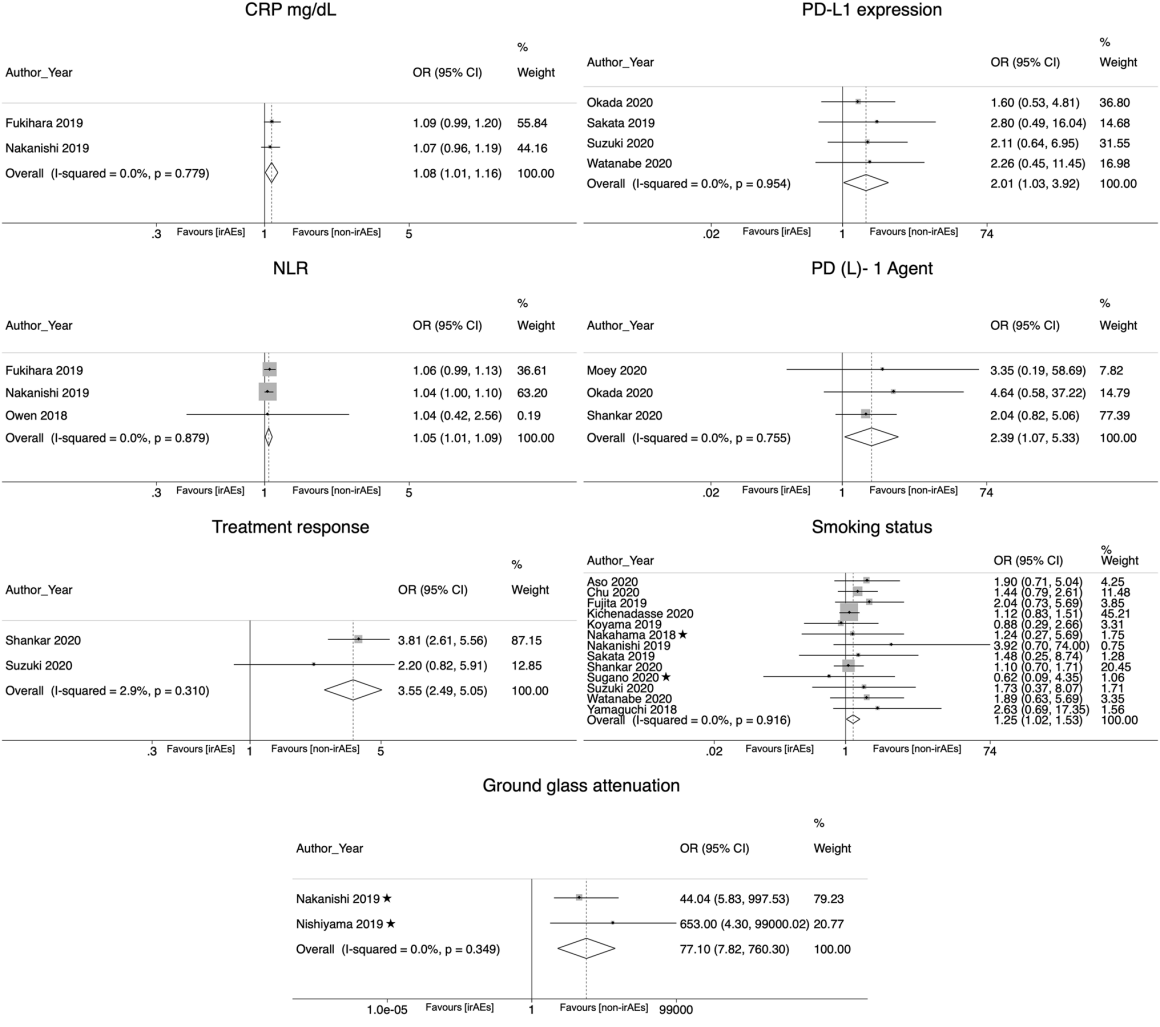
Forest plots for random effect meta-analysis of the associations between potential risk factors and immune-related adverse events. *OR* odds ratio; *CI* confidence interval; *CRP* C-reactive protein; *NLR* neutrophil–lymphocyte ratio. ✭Studies that provided adjusted odds ratios

In the meta-analyses combining the crude and adjusted ORs, the following risk factors were significantly associated with the development of irAEs: C-reactive protein (OR 1.08; CI 95% 1.007–1.158); neutrophil lymphocyte ratio (NLR) (OR 1.046; CI 95% 1.006–1.088), the presence of ground glass attenuation in CT imaging (OR 77.1; CI 95% 7.82–760.3), use of PD-1 inhibitor agent (OR 2.39, CI 95% 1.073—5.326), higher PD-L1 expression (OR 2.009, CI 95% 1.03–3.921), an active or former smoking status (OR 1.249; CI 95% 1.021–1.528), and a better treatment response (OR 3.548; CI 95% 2.49–5.055) (Table 2; Fig. 1).

**Table 2:** Random effects meta-analysis. Pooled crude and adjusted odds ratios reflecting the association between risk factors and irAEs in patients treated with ICIs

Risk factor	Pooled OR	LCI	UCI	Chi-2	Chi-2 *p* value	I2	*P* value	No. of studies	No. of participants
Abnormal CT findings (abnormal CT vs normal CT)	1.193	0.607	2.343	1.04	0.307	4.20%	0.609	2 [29] [48]	253
Age (≥ 65 vs < 65)	1.124	0.904	1.396	11.43	0.178	30%	0.292	10 [25] [26] [27] [28] [31] [33] [45] [47] [53] [55]	2442
Age (years)	0.988	0.954	1.023	0.39	0.533	0.00%	0.49	2 [29] [48]	253
Asthma (history of asthma vs no history of asthma)	1.729	0.226	13.209	0.87	0.35	0.00%	0.598	2 [26] [56]	305
Black ethnicity (black vs white ethnicity)	0.709	0.346	1.456	1.42	0.233	29.50%	0.349	2 [51] [55]	401
COPD (history of COPD vs no history of COPD)	1.439	0.983	2.108	5.21	0.391	3.90%	0.061	6 [26] [29] [32] [48] [51] [56]	853
CRP (mg/dL)	1.08	1.007	1.158	0.08	0.779	0.00%	0.03	2 [29] [48]	253
Diabetes (history of diabetes vs no history of diabetes)	1.672	0.86	3.248	2.8	0.247	28.50%	0.129	3 [26] [51] [56]	501
Ground glass attenuation (ground glass attenuation vs normal CT)	77.098	7.818	760.304	0.88	0.349	0.00%	0.0001	2 [28] [29]	131
Histology (squamous vs non-squamous)	0.934	0.806	1.082	23.33	0.077	35.70%	0.363	16 [25–27, 29–33, 46–49, 53, 54, 56, 58]	5155
Hypertension (history of hypertension vs no history of hypertension)	0.508	0.247	1.048	0.52	0.471	0.00%	0.067	2 [51, 56]	334
ILD (previous history of ILD vs no history of ILD)	1.174	0.479	2.876	1.41	0.493	0.00%	0.726	3 [26, 32, 48]	439
LDH (≥ 240 vs < 240 U/L)	1	0.999	1.001	7.03	0.071	57.30%	0.749	4 [29, 30, 33, 49]	1955
NLR (≥ 5 vs < 5)	1.046	1.006	1.088	0.26	0.879	0.00%	0.026	3 [29] [48] [52]	344
PD-(L)1 agent (PD-1 vs PD-L1 inhibitor)	2.39	1.073	5.326	0.56	0.755	0.00%	0.033	3 [32] [51] [54]	921
PD-L1 expression (≥ 50% vs < 50%)	2.009	1.03	3.921	0.33	0.954	0.00%	0.041	4 [32] [53] [56] [58]	513
PD-L1 expression (positive vs negative)	0.996	0.791	1.254	0	0.991	0.00%	0.973	2 [25] [49]	1680
Pembrolizumab treatment (pembrolizumab treatment vs other ICIs)	1.586	0.934	2.693	8.45	0.037	64.50%	0.088	4 [48] [53] [55] [58]	648
Performance status (≥ 2 vs 0–1)	0.801	0.655	0.979	25.3	0.088	32.80%	0.055	18 [25–33, 45–49, 53, 54, 56, 58]	5488
Preexisting anti-nuclear antibodies (preexisting anti-nuclear antibodies vs no preexisting anti-nuclear antibodies)	1.524	0.857	2.709	1.54	0.215	35.00%	0.151	2 [27] [46]	455
Previous cardiovascular disease (history of cardiovascular disease vs no history of cardiovascular disease)	2.295	0.93	5.663	2.26	0.133	55.70%	0.072	2 [51] [56]	334
Previous cerebrovascular disease (history of cerebrovascular disease vs no history of cerebrovascular disease)	0.905	0.277	2.959	0.76	0.383	0.00%	0.869	2 [51] [56]	334
Previous radiation pneumonitis (history of radiation pneumonitis vs no history of radiation pneumonitis)	2.584	0.971	6.877	3.52	0.061	71.60%	0.057	2 [29] [30]	284
Radiotherapy (previous radiotherapy vs no radiotherapy)	1.04	0.715	1.511	1.5	0.96	0.00%	0.839	7 [26] [29] [31] [32] [48] [51] [52]	939
Sex (male vs female)	1.033	0.904	1.182	23.15	0.185	22.20%	0.631	19 [25] [26] [27] [28] [29] [30] [31] [32] [33] [34] [46] [47] [48] [49] [51] [53] [54] [56] [58]	5630
Smoking pack-years (≥ 50 vs < 50 packs/year)	0.994	0.981	1.007	0.12	0.726	0.00%	0.368	3 [28] [32] [48]	302
Smoking status (current/former vs never smoker)	1.249	1.021	1.528	6	0.916	0.00%	0.031	13 [25] [26] [27] [29] [30] [31] [33] [46] [49] [53] [54] [56] [58]	3873
Stage (stage III/IV vs I/II)	1.269	0.846	1.901	17.02	0.009	64.80%	0.249	7 [26] [32] [51] [53] [54] [56] [58]	1499
Combination treatment (ICI + chemotherapy vs monotherapy)	0.686	0.439	1.073	5.4	0.067	62.90%	0.099	3 [27] [54] [55]	1128
Treatment line (first vs second/third line)	1.039	0.792	1.363	6.64	0.467	0.00%	0.782	8 [27] [32] [33] [48] [53] [58] [56] [49]	2654
Treatment response (complete response/partial response/stable disease vs progressive disease)	3.548	2.49	5.055	1.03	0.31	2.90%	0.0001	2 [54] [56]	761
Use of steroids (previous use of steroids vs no previous use of steroids)	1.58	0.976	2.556	12.17	0.002	83.60%	0.063	3 [26] [27] [51]	663
White blood cell count (**≥ **10^3^ vs < 10^3^ /mm^3^)	1	1	1	3.84	0.05	74.00%	0.35	2 [29] [48]	253

*OR* odds ratio; *LCI* lower confidence interval; *UCI* upper confidence interval; *COPD* chronic obstructive pulmonary disease; *CRP* C-reactive protein; *ILD* interstitial lung disease; *NLR* neutrophil–lymphocyte ratio; *PD-L1* programmed death-ligand 1; *LDH* lactate dehydrogenase; *U/L* units per liter; *mg/dL* milligrams per deciliter

In the sensitivity analyses excluding the crude ORs, 10 studies were included [25–34]. Risk factors that could be pooled and were significantly related with the development of irAEs were squamous histology (OR 1.847; CI 95% 1.048–3.256), a higher number of pack-years of smoked tobacco (OR 2.662; CI 95% 1.014–6.989) and the presence of ground glass attenuation in CT imaging (OR 77.1; CI 95% 7.82–760.3) (Supplementary Table 2).

As part of the stratified meta-analyses by type of irAE (Supplementary Table 3), it was observed that men compared to women are at higher risk of developing ILD (OR 1.79; CI 95% 1.054–3.041). In the case of pneumonitis, no significant associations were found with the tested risk factors (Supplementary Table 3). For irAEs classified as severe, patients with a worse ECOG performance status are at higher risk (OR 4.274; CI 95% 1.215–14.925; Supplementary Table 3).

For risk factors with significant heterogeneity (*I*^*2*^ > 50%), additional analyses stratified by quality/risk of bias and region were performed to explore sources of heterogeneity. For stage and combination treatment (ICI + chemotherapy vs. monotherapy), quality/risk of bias of included studies was an explanatory factor for the observed heterogeneity in the pooled analyses. For these risk factors, there was no longer heterogeneity when only moderate or high quality studies were pooled (Supplementary Table 4). For the other stratified analysis by study region, we found that region can be a source of heterogeneity for stage as a risk factor and the use of pembrolizumab. Significant heterogeneity was no longer observed for these risk factors when only studies from Asia were pooled (Supplementary Table 5).

Multiple risk factors were only reported by a single study, rendering them unsuitable for meta-analysis. The following risk factors were found to be statistically significantly related to the development of irAEs in one of the studies: a decrease in albumin level, the absence of concomitant irAEs a higher eosinophil count, a higher fibrosis score, imaging findings of airway obstruction adjacent to lung tumors (IAOT), a lower lung immune prognostic index, the presence of less than three organs affected by the cancer, the history of elevated autoantibodies, history of preexisting rheumatoid factor, history of previous interstitial pneumonia, a higher tumor burden, use of beta-blockers, use of loop diuretics, and use of taxanes (Supplementary Table 1). Finally, some studies reported risk factors for specific types of irAEs as a secondary outcome. In these studies, preexisting rheumatoid factor increased the risk of developing skin reactions, and women reported to be at a higher risk of developing endocrinopathies (Supplementary Table 6).

## Discussion

In this meta-analysis including 25 studies and 6696 patients, several risk factors for the development of irAEs in patients with NSCLC treated with ICIs were identified. These factors were increased C-reactive protein level, increased NLR, ground glass attenuation on CT, use of PD-1 inhibitor therapy, PD-L1 expression, smoking, and treatment response. These risk factors are mostly related to the inflammatory response or the treatment response. This illustrates that a predisposition to acute inflammatory response and/or poorer immune self-tolerance may be the underlying mechanisms explaining why the presence of the identified factors increases the risk of irAEs following treatment with ICIs in NSCLC patients.

First of all, the laboratory markers of inflammatory response were shown to be associated with a higher risk of irAEs. C-reactive protein and NLR are both markers of ongoing inflammatory processes, and stimulation of the immune system by ICIs can then further promote the immune response resulting in irAEs [35–37].

With regard to treatment and response, the identified risk factors are in line with what can be expected based on previous literature. The relationship between a better response to treatment and a greater occurrence of irAEs has been previously reported [38]. Related to this, a higher PD-L1 expression, a risk factor studied in this systematic review, has previously been shown to be associated with better treatment response [39]. As for the type of immune checkpoint targeted by ICIs, PD-1 inhibitors have been shown to have a worse safety profile than PD-L1 inhibitors [40].

Moreover, smoking was shown to be associated with a higher risk of irAEs, likely due to its impact on inflammation in the airway and lung parenchyma. Smoking is known to cause changes in the patterns of the normal immune response and inflammatory processes, as well as recruitment of autoantibodies [41, 42]. This enhancement in inflammatory response and recruitment of autoantibody-stimulated immune response cells in lung tissue can lead to a loss of self-tolerance and may contribute to the development of irAEs.

Furthermore, the presence of ground glass attenuation visible on CT images showed an association with patient’s risk of irAEs. Ground glass attenuation can be a marker of pre-existing inflammatory activity and susceptibility to acute inflammatory response, that can further be enhanced by ICIs [29] [43].

Several of the risk factors presented in this review and meta-analysis are assessed as part of routine care in patients before the initiation of immunotherapy and/or as part of their follow-up. Some of these risk factors markedly increased the risk of developing irAEs, such as the presence of ground glass attenuation in CT. A favorable treatment response tripled the risk of irAEs. The use of PD-1 inhibitors as opposed to PD-L1 inhibitors, and a high expression of PD-L1 doubled the risk of irAEs. Other risk factors had a modest effect on the risk of irAEs, such as a history of smoking, high NLR and CRP values.

### Strengths and limitations

The major strength of this study is the high level of homogeneity among studies; all studies shared the same study design and had similar patient inclusion criteria. Furthermore, in line with previous studies we found that overall, irAEs of interest were reported for 25% of the patients. However, since many studies focused on selected irAEs only (i.e., pneumonitis or interstitial lung disease), it is likely that this estimate is an underestimation of the percentage of patients experiencing irAEs of any type.

A main limitation of this study is that some risk factors may remain unexplored, since in several cases only one paper explored a specific risk factor, making it not possible to pool the results in the meta-analysis. Some of these single study-based risk factors excluded from the meta-analysis share a possible causal origin in the alterations of the immune system, such as a history of high levels of antibodies or rheumatoid factor. In the case of ground glass attenuation as a risk factor, the results should be interpreted with caution, since the study carried out by Nishiyama includes only patients with a history of having preexisting ILD, which could lead to a selection bias. Another limitation is that in a substantial proportion of the cases we extracted crude OR from the reported frequencies and as such we could not take potential confounding into account. For this, we also conducted analyses excluding these crude ORs, which lowered the number of risk factors that could be analyzed. All findings were comparable, with the exception of squamous histology of the tumor, being significant in the adjusted analysis only. Likewise, in the analyses stratified by type of irAE, we found that a worse performance status is a risk factor for developing severe irAEs, while the opposite was true for the main meta-analysis pooling studies irrespective of the type of irAE they reported as outcome.

Finally, another important aspect to take into account is the observed heterogeneity after pooling the results for some risk factors. In our sensitivity analysis, we stratified by region and study quality/risk of bias as assessed with QUIPS and found that region can explain part of the heterogeneity in the effect of stage as well as the use of pembrolizumab, and quality/risk of bias can contribute to heterogeneity in the case of stage and combination treatment. In these cases, heterogeneity (*I*^*2*^) decreased below 50% when stratified by region or quality/risk of bias.

### Implications and recommendations

The risk factors identified in this review may help in selecting treatment regimen for patients at a higher risk of developing irAEs and could contribute to the decision of starting therapy with ICIs. When deciding to treat identified patients with a higher risk for irAEs, these patients should be monitored more closely. Early, adequate treatment of irAEs will result in a better clinical outcome [44]. Given the limited number of studies looking at the relationship between patient characteristics and the risk of irAEs, future studies are needed to explore determinants of irAEs and search for new mechanisms through which these risk factors contribute to the development of irAEs.

## Supplementary Information

Below is the link to the electronic supplementary material.

Supplementary file1 (PDF 25 KB)

Supplementary file2 (PDF 142 KB)

Supplementary file3 (PDF 638 KB)

## Data Availability

The authors confirm that the data supporting the findings of this study are available within the supplementary materials.
