# Supplementary material for: Risk factors for adverse events induced by immune checkpoint inhibitors in patients with non-small-cell lung cancer: a systematic review and meta-analysis
Source: Cancer Immunol Immunother. 2021 Jun 30;70(11):3069–80. doi: 10.1007/s00262-021-02996-3 (PMC8505368; doi:10.1007/s00262-021-02996-3)
Supplement: Supplementary file 1 — Supplementary file1 (PDF 25 KB) [file 262_2021_2996_MOESM1_ESM.pdf]

Supplementary material 1. Search strategy

Pubmed	(**lung neoplasms**[Mesh] OR **lung cance***[tiab] OR **lung neoplas***[tiab])<br>AND<br>("**Programmed Cell Death 1 Receptor/antagonists and inhibitors**"[Mesh] OR **PD-1**[tiab] OR **PD1**[tiab] OR **PD-L1**[tiab] OR **CTLA4**[tiab] OR **CTLA-4**[tiab] OR **cytotoxic T-lymphocyte-associated protein 4**[tiab] OR **PDL1**[tiab] OR **Programmed Cell Death 1***[tiab] OR **Immune Checkpoint***[tiab] OR **nivolumab**[tiab] OR **pembrolizumab**[tiab] OR **atezolizumab**[tiab] OR **durvalumab**[tiab] OR **cemiplimab**[tiab] OR **avelumab**[tiab] OR **monoclonal antibodies** [tiab] OR **ipilimumab**[tiab] OR **tremelimumab**[tiab])<br>AND<br>(**drug related side effects and adverse reactions**[Mesh] OR **adverse reactio***[tiab] OR **adverse even*[**tiab] OR **side effec***[tiab])<br>AND<br>(**risk factor**[Mesh] OR **risk facto***[tiab] OR **predicto***[tiab] OR **exposur***[tiab])<br>AND<br>("2000/01/01"[Date - Publication] : "2020/11/12"[Date - Publication])
Embase	('**lung tumor**'/exp OR '**lung tumor**':ab,ti OR '**lung cancer**':ab,ti OR '**lung neoplasm***':ab,ti)<br>AND<br>('**pdl1 gene'**/exp OR **'immune checkpoint inhibitor'**/exp OR **'immunomodulating agent'**/exp OR '**immunomodulating agent**':ab,ti OR '**avelumab**':ab,ti OR '**cemiplimab**':ab,ti OR '**durvalumab**':ab,ti OR '**atezolizumab**':ab,ti OR '**pembrolizumab**':ab,ti OR '**nivolumab**':ab,ti OR '**pdl1**':ab,ti OR '**pd1**':ab,ti OR '**immune checkpoint inhibitor**':ab,ti OR '**tremelimumab**':ab,ti OR ' **ipilimumab**':ab,ti OR **'ctla4'**:ab,ti OR **'ctla**-**4'**:ab,ti OR '**cytotoxic T-lymphocyte-associated protein 4**':ab,ti)<br>AND<br>('**side effect**'/exp OR '**side effect**':ab,ti OR '**adverse drug reaction**'/exp OR '**adverse drug reaction**':ab,ti OR '**adverse event**'/exp OR '**adverse event**':ab,ti)<br>AND<br>('**risk factor**'/exp OR '**risk factor**':ab,ti OR **'exposure'**/exp OR **exposure**:ab,ti OR **predicto***:ab,ti)<br>AND<br>[article]/lim AND [embase]/lim NOT ([embase]/lim AND [medline]/lim) AND [2000-2020]/py
Web of science	TS=("**lung neoplasms**" OR "**lung cance***" OR "**lung neoplas***")<br>AND<br>TS= ("**Immune Checkpoint Inhibit***" OR "**PD-1**" OR "**PD-L1**" OR "**PD1**" OR "**PDL1**" OR "**avelumab**" OR "**cemiplimab**" OR "**durvalumab**" OR "**atezolizumab**" OR "**pembrolizumab**" OR "**nivolumab**" OR "**CTLA-4**" OR "**CTLA4**" OR "'**tremelimumab**" OR " **ipilimumab**" OR " **cytotoxic T-lymphocyte-associated protein 4**")<br>AND<br>TS= ("**drug related side effects**" OR "**adverse reactio***" OR "**adverse even***" OR "**side effec***")<br>AND<br>TS= ("**risk facto***" OR "**predicto***" OR "**exposur***")
