# Supplementary material for: Risk factors for adverse events induced by immune checkpoint inhibitors in patients with non-small-cell lung cancer: a systematic review and meta-analysis
Source: Cancer Immunol Immunother. 2021 Jun 30;70(11):3069–80. doi: 10.1007/s00262-021-02996-3 (PMC8505368; doi:10.1007/s00262-021-02996-3)
Supplement: Supplementary file 2 — Supplementary file2 (PDF 142 KB) [file 262_2021_2996_MOESM2_ESM.pdf]

Supplementary material 2. Data collection form

THE COCHRANE COLLABORATION®

Data collection form

Intervention review – RCTs and non-RCTs

**Review title or ID**

**Study ID** *(surname of first author and year first full report of study was published e.g. Smith 2001)*

General Information

1. **Date form completed** *(dd/mm/yyyy)*	
2. **Name/ID of person extracting data**	
3. **Study funding source** *(including role of funders)*	

Eligibility

**Study Characteristics**	**Review Inclusion Criteria**	**Yes/ No / Unclear**	**Location in text**
4. **Type of study**	Randomised trial		
	Non-randomised trial		
5. **Participants**			
6. **Types of Exposure**			
7. **Types of outcome measures**			
8. **Decision:**			
9. **Reason for exclusion**			

DO NOT PROCEED IF STUDY EXCLUDED FROM REVIEW

Population and setting

	**Description** *Include comparative information for each group (i.e. intervention and controls) if available*	**Location in text** *(pg & ¶/fig/table)*
10. **Population description** *(from which study participants are drawn)*		
11. **Setting** *(including location and social context)*		
12. **Inclusion criteria**		
13. **Exclusion criteria**		

Data collection form

Intervention review – RCTs and non-RCTs

Methods

	Descriptions as stated in report/paper	Location in text *(pg & ¶/fig/table)*
14. **Aim of study**		
15. **Design** *(e.g. parallel, crossover, non-RCT)*		
16. **Start date**		
17. **End date**		
18. **Duration of participation** *(from recruitment to last follow-up)*		

Participants

Provide overall data and, if available, comparative data for each intervention or comparison group. Add as many rows as needed

**Demographics**	**Total**		**Group 1**		**Group 2**	
	**N**	**%**	**N**	**%**	**N**	**%**
**Age**						
**Male**						
**Female**						
**Type of cancer**						
**Other important demographics**						
**Other treatment**						

Outcomes

Copy and paste table for each outcome.

Outcome 1

	Description as stated in report/paper		Location in text
19. **Outcome definition** *(with diagnostic criteria if relevant)*			
20. **Person measuring/ reporting**			
21. **Is outcome/tool validated?**	*Yes/No/Unclear*		

Results

Copy and paste the appropriate table for each outcome, including additional tables for each time point and subgroup as required.

For randomised or non-randomised trial - Dichotomous outcome

	**Description as stated in report/paper**				**Location in text**
22. **Comparison**					
23. **Outcome**					
24. **Subgroup**					
25. **Results** *Note whether: post-intervention OR change from baseline And whether Adjusted OR Unadjusted*	**Intervention**		**Comparison**		
	No. events	No. participants	No. events	No. participants	
26. **Baseline data**	**Intervention**		**Comparison**		

	Description as stated in report/paper				Location in text
	No. events	No. participants	No. events	No. participants	
27. **Statistical methods used and appropriateness of these methods**					

Other information

	Description as stated in report/paper	Location in text
28. **Key conclusions of study authors**		
