# Supplementary material for: Risk factors for adverse events induced by immune checkpoint inhibitors in patients with non-small-cell lung cancer: a systematic review and meta-analysis
Source: Cancer Immunol Immunother. 2021 Jun 30;70(11):3069–80. doi: 10.1007/s00262-021-02996-3 (PMC8505368; doi:10.1007/s00262-021-02996-3)
Supplement: Supplementary file 3 — Supplementary file3 (PDF 638 KB) [file 262_2021_2996_MOESM3_ESM.pdf]

Supplementary material 3

Supplementary Figure 1. Flowchart of study selection and design

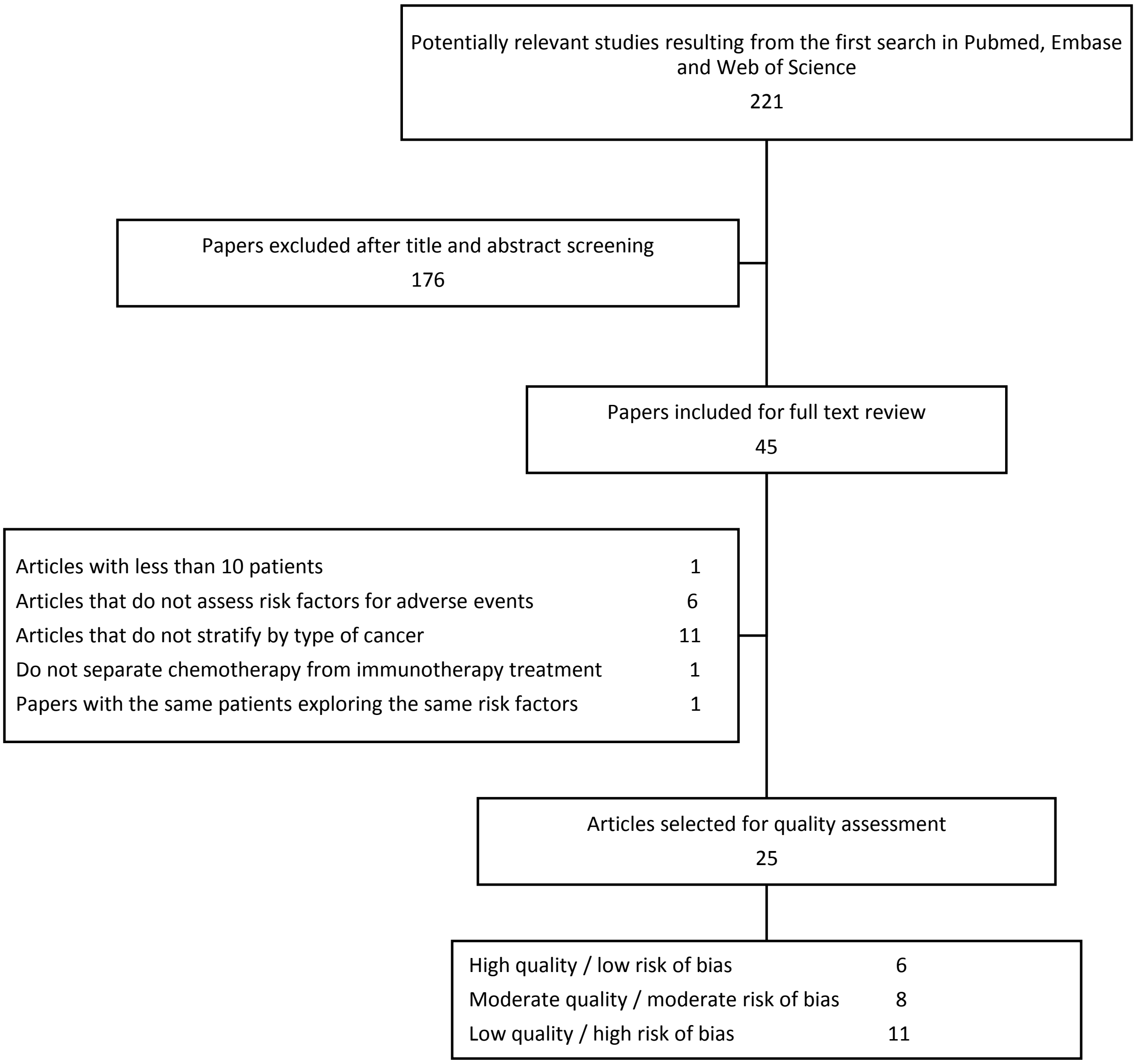

Supplementary Figure 2. QUIPS Quality assessment of the studies included to the meta-analysis.

	1. Study Participation	2. Study Attrition	3. Prognostic factor measurement	4. Outcome Measurement	5. Study Confounding	6. Statistical analysis and reporting	Overall evaluation
Ahmed 2020	L	M	L	M	H	M	H
Aso 2020	L	M	L	L	H	M	M
Chu 2020	L	M	L	L	L	L	L
Cortellini 2020	L	M	M	L	H	H	H
Duma 2019	L	M	L	L	L	L	L
Fujita 2019	L	M	M	L	H	L	M
Fukihara 2019	L	M	M	L	L	L	L
Kichenadasse 2020	L	M	M	L	M	H	H
Koyama 2019	L	M	L	L	L	L	L
Metro 2020	L	M	L	M	H	M	H
Moey 2020	L	M	M	L	H	H	H
Nakahama 2018	L	H	L	M	L	L	M
Nakanishi 2019	L	H	L	L	L	L	M
Nishiyama 2019	L	M	M	M	H	H	H
Okada 2020	L	M	L	L	L	L	L
Owen 2018	L	M	L	L	H	H	H
Sakata 2019	L	M	L	L	M	H	H
Shankar 2020	L	M	L	L	H	H	H
Sugano 2020	L	M	L	L	M	L	M
Suresh 2018	L	M	L	L	L	L	L
Suzuki 2020	M	M	L	L	M	L	M
Toi 2019	L	M	L	L	M	H	H
Watanabe 2020	L	M	L	L	H	M	M
Yamaguchi 2018	L	M	L	L	M	L	M
Zeng 2020	L	M	L	L	H	H	H

Green/L (low risk of bias), yellow/M (moderate risk of bias), red/H (high risk of bias)

Supplementary Table 1. Classification of crude and adjusted odds rations reflecting the association between risk factors and irAEs in patients treated with ICIs per publication.

Author/Year	Risk factor	Type of irAE	OR	LCI	UCI	p Value	Analysis
Fukihara 2019	Abnormal CT findings	Pneumonitis	1.53	0.669	3.5	0.313	Crude
Nakanishi 2019		ILD	0.725	0.225	2.334	0.585	Crude
Ahmed 2020	Age (≥ 65 vs <65 years old)	irAEs	2.155462	0.994621	4.671143	0.0636	Crude
Chu 2020		Pneumonitis	1.417	0.789	2.746	0.225	Adjusted
Cortellini 2020		irAEs	1.041	0.800	1.353	0.789	Crude
Fujita 2019		Infection diseases	1.53	0.48	5.47	0.48	Adjusted
Koyama 2019		immune-related thyroid dysfunction	0.23	0.03	1.86	0.168	Adjusted
Nishiyama 2019		Acute exacerbation of Interstitial lung disease	0.75187	0.082644	6.6666	0.8	Adjusted
Sakata 2019		Severe irAEs	0.676923	0.114453	4.00361	1	Crude
Sugano 2020		ILD	0.223714	0.044053	1.136364	0.07	Adjusted
Suresh 2018		Pneumonitis	1	1	1	0.69	Crude
Yamaguchi 2018		Pneumonitis	2.15	0.71	8.01	0.18	Crude
Fukihara 2019	Age (years)	Pneumonitis	0.98	0.939	1.023	0.347	Crude
Nakanishi 2019		ILD	1.003	0.949	1.068	0.919	Crude
Fukihara 2019	Albumin	Pneumonitis	0.381	0.179	0.808	0.012	Adjusted
Owen 2018	ALI	irAEs	2.042857	0.8128516	5.134105	0.1547	Crude
Fujita 2019	ALK gene	Infection diseases	1.2	0.77	5.85	0.56	Adjusted
Fukihara 2019	ALT	Pneumonitis	1.017	0.992	1.041	0.185	Crude
Fukihara 2019	AST	Pneumonitis	0.987	0.949	1.028	0.538	Crude
Fujita 2019	Asthma	Infection diseases	4.322581	0.2630676	71.02625	0.3474	Crude
Suzuki 2020		ILD	0.620596206	0.03217908	11.9686332	0.76484552	Crude
Suzuki 2020	Best supportive care after ICIs	ILD	0.8918919	0.3383415	2.351089	0.8082	Crude
Moey 2020	Black ethnicity	Inhibitor-Related Cardiotoxicity	0.4503367	0.1596178	1.270555	0.1664	Crude
Suresh 2018		Pneumonitis	1.08	0.37	2.72	0.87	Crude
Metro 2020	Brain metastasis	irAEs	1.05913	0.409498	2.739347	1	Crude
Fujita 2019	Bronchiectasis	Infection diseases	0.5615764	0.1566791	2.012827	0.575	Crude
Zeng 2020	Charlson comorbidity index	irAEs	1.555555659	4.47995957	0.54012829	0.431	Crude
Shankar 2020	Combination ICI treatment	irAEs	0.8148148	0.3476475	1.909759	0.8336	Crude
Moey 2020	Concomitant irAEs	Inhibitor-Related Cardiotoxicity	0.0582524	0.0174364	0.1946124	0	Crude
Fujita 2019	COPD	Infection diseases	1.425	0.6368462	3.188564	0.4016	Crude
Fukihara 2019		Pneumonitis	1.837798	0.800669	4.218346	0.18923	Crude
Moey 2020		Inhibitor-Related Cardiotoxicity	1.68306	0.7012057	4.039744	0.2565	Crude
Nakanishi 2019		ILD	0.977	0.275	3.161	0.971	Crude
Okada 2020		ILD	2.657143	0.9509449	7.424624	0.0913	Crude
Suzuki 2020		ILD	0.5828571	0.2093999	1.622362	0.3354	Crude
Fujita 2019	Corticosteroids during/after nivolumab	Infection diseases	1.374863	0.63491	2.977193	0.437459	Crude
Fukihara 2019	Creatinine	Pneumonitis	1.356	0.414	4.445	0.615	Crude
Yamaguchi 2018	CRP (≥1mg vs <1mg)	Pneumonitis	0.67	0.23	1.85	0.44	Crude
Nakahama 2018	CRP (3mg vs >3mg)	ILD	0.95	0.3	2.95	0.925	Adjusted

Fukihara 2019	CRP (mg/dl)	Pneumonitis	1.09	0.993	1.197	0.071	Crude
Nakanishi 2019		ILD	1.06826	0.96172	1.1866	0.236	Crude
Fujita 2019	Diabetes	Infection diseases	3.61	1.14	11.4	0.028	Adjusted
Moey 2020		Inhibitor-Related Cardiotoxicity	1.28267	0.4951607	3.322645	0.6172	Crude
Suzuki 2020		ILD	0.8253968	0.1727878	3.942871	1	Crude
Suzuki 2020	Digestive ulcer	ILD	2.074074	0.3881364	11.08318	0.3268	Crude
Nakanishi 2019	Driver mutation	ILD	0.454	0.024	2.699	0.43	Crude
Fujita 2019	EGFR mutation	Infection diseases	0.572391	0.203977	1.606212	0.353521	Crude
Koyama 2019	EGFR/ALK positive	immune-related thyroid dysfunction	0.43	0.05	3.47	0.426	Crude
Yamaguchi 2018	Emphysema score	Pneumonitis	0.68	0.16	2.73	0.58	Adjusted
Chu 2020	Eosinophil count	Pneumonitis	3.518	1.851	6.686	0.001	Adjusted
Yamaguchi 2018	Fibrosis	Pneumonitis	9.53	2.47	44.79	0.0008	Adjusted
Nakanishi 2019	Ground glass attenuation	ILD	44.04	5.83	997.528	0.001	Adjusted
Nishiyama 2019		Acute exacerbation of Interstitial lung disease	653	4.3	99000	0.01	Adjusted
Suzuki 2020	Hepatic disease	ILD	1.189474	0.1316201	10.74948	1	Crude
Aso 2020	Histology (Squamous vs non-squamous)	skin reactions	1.121528	0.5587607	2.251097	0.8584	Crude
Chu 2020		Pneumonitis	1.430	0.759	2.693	0.3113	Crude
Cortellini 2020		irAEs	0.882	0.647	1.202	0.4368	Crude
Fujita 2019		Infection diseases	0.865455	0.343016	2.183603	0.822459	Crude
Fukihara 2019		Pneumonitis	0.76	0.311	1.857	0.547	Crude
Kichenadasse 2020		irAEs	0.8124363	0.6297399	1.048136	0.1129	Crude
Koyama 2019		immune-related thyroid dysfunction	0.52	0.14	1.91	0.323	Crude
Nakahama 2018		ILD	1.28	0.44	3.78	0.648	Adjusted
Nakanishi 2019		ILD	1.609	0.323	6.327	0.529	Crude
Okada 2020		ILD	1.923077	0.5907563	6.260153	0.3184	Crude
Sakata 2019		Severe irAEs	6.25	1.03691	37.67204	0.0772	Crude
Shankar 2020		irAEs	0.7762647	0.5256311	1.146407	0.2087	Crude
Sugano 2020		ILD	1.234568	0.3508772	4.347826	0.75	Adjusted
Suzuki 2020		ILD	0.2484994	0.0690348	0.8945047	0.0256	Crude
Watanabe 2020		ILD	2.276786	1.080531	4.797413	0.0314	Crude
Yamaguchi 2018		Pneumonitis	2	0.63	5.81	0.23	Crude
Fujita 2019	History of thoracic surgery	Infection diseases	0.9230769	0.3454827	2.46632	1	Crude
Nakanishi 2019	Honeycombing in CT	ILD	1.932	0.037	83.463	0.729	Adjusted
Moey 2020	Hyperlipidemia	Inhibitor-Related Cardiotoxicity	0.4398496	0.1429029	1.35384	0.2271	Crude
Moey 2020	Hypertension	Inhibitor-Related Cardiotoxicity	0.6093366	0.2539677	1.461962	0.3585	Crude
Suzuki 2020		ILD	0.3441176	0.0951813	1.24412	0.1191	Crude
Fujita 2019	Hypothyroidism	Infection diseases	0.5898618	0.0699781	4.97208	1	Crude
Nakahama 2018	IAOT	ILD	6.59	1.82	23.9	0.004	Adjusted
Fujita 2019	ILD	Infection diseases	0.5120968	0.0617382	4.247662	1	Crude
Fukihara 2019		Pneumonitis	1.898551	0.563179	6.400268	0.288898	Crude
Okada 2020		ILD	0.78	0.14	4.24	0.77	Adjusted
Okada 2020		Severe irAEs	0.72	0.075	6.88	0.77	Adjusted
Nakanishi 2019	KL-6	ILD	0.99975	0.9986	1.0001	0.297	Crude
Owen 2018	KRAS mutation	irAEs	0.3320158	0.1019756	1.080989	0.0766	Crude
Kichenadasse 2020	LDH	irAEs	0.811978	0.641261	1.028143	0.085	Crude

Nakahama 2018		ILD	2.1	0.81	5.38	0.126	Adjusted
Nakanishi 2019		ILD	0.99987	0.99908	1.0007	0.659	Crude
Yamaguchi 2018		Pneumonitis	0.45	0.12	1.34	0.16	Crude
Kichenadasse 2020	Lung immune prognostic index	irAEs	1.650946	1.119867	2.433882	0.0098	Crude
Nakanishi 2019	Lymphocyte	ILD	0.99933	0.99834	1.0003	0.17	Crude
Nakanishi 2019	Neutrophil count	ILD	1.00003	0.99999	1.0001	0.125	Crude
Fukihara 2019	NLR	Pneumonitis	1.06	0.993	1.131	0.08	Crude
Nakanishi 2019		ILD	1.03783	0.99754	1.1014	0.066	Crude
Owen 2018		irAEs	1.035714	0.4191255	2.559386	1	Crude
Fujita 2019	No. Cycles	Infection diseases	1.76	0.6	5.53	0.31	Adjusted
Shankar 2020	Non-white ethnicity	irAEs	1.072435	0.7228447	1.591099	0.7616	Crude
Cortellini 2020	Number of organs affected	irAEs	0.553	0.424	0.721	0.0001	Crude
Suresh 2018	Other treatments	Pneumonitis	0.19	0.01	1	0.11	Crude
Moey 2020	PD-(L)1 Agent (PD-1 vs PD-L1 inhibitors)	Inhibitor-Related Cardiotoxicity	3.346749226	0.1908319	58.6942251	0.41606877	Crude
Okada 2020		ILD	4.636364	0.5774652	37.22453	0.1827	Crude
Shankar 2020		irAEs	2.035623	0.8188542	5.06044	0.1628	Crude
Nakanishi 2019	PD-L1 expression (≥1%/<1%)	ILD	0.686	0.065	7.235	0.754	Crude
Okada 2020	PD-L1 expression (≥50% vs. <50%)	ILD	1.596059	0.53013	4.805245	0.4195	Crude
Sakata 2019		Severe irAEs	2.8	0.488907	16.035754	0.431511	Crude
Suzuki 2020		ILD	2.113095	0.6426302	6.948275	0.3024	Crude
Watanabe 2020		ILD	2.26087	0.4463522	11.45179	0.4816	Crude
Kichenadasse 2020	PD-L1 expression (positive vs. negative)	irAEs	0.9963412	0.7871292	1.26116	1	Crude
Koyama 2019		immune-related thyroid dysfunction	0.99	0.33	2.98	0.983	Crude
Fukihara 2019	Pembrolizumab treatment (Pembrolizumab vs other ICIs)	Pneumonitis	3.259	1.361	7.807	0.008	Adjusted
Sakata 2019		Severe irAEs	3.2	0.731794	13.993012	0.180373	Crude
Suresh 2018		Pneumonitis	0.344916	0.077318	1.538675	0.175965	Crude
Watanabe 2020		ILD	1.028085	0.4346893	2.431527	1	Crude
Ahmed 2020	Performance status (≥2 vs 0-1)	irAEs	0.9161491	0.4247466	1.97607	0.8465	Crude
Aso 2020		Skin reactions	0.4803922	0.06571	3.512045	0.5985	Crude
Chu 2020		Pneumonitis	2.427184466	0.55157198	10.6382979	0.241	Crude
Cortellini 2020		irAEs	2.02963	1.35978	3.0846	0.0003	Crude
Fujita 2019		Infection diseases	0.4975418	0.203137	1.218625	0.1336	Crude
Fukihara 2019		Pneumonitis	0.569	0.205	1.583	0.28	Crude
Kichenadasse 2020		irAEs	0.880726705	0.1770669	4.38071382	1	Crude
Koyama 2019		immune-related thyroid dysfunction	1.960784314	0.41666667	9.09090909	0.399	Crude
Nakahama 2018		ILD	1.587301587	0.45248869	5.55555556	0.471	Adjusted
Nakanishi 2019		ILD	1.017	0.229	7.159	0.984	Crude
Nishiyama 2019		Acute exacerbation of Interstitial lung disease	1.028571	0.104442	10.129603	1	Crude
Okada 2020		ILD	0.290697674	0.08474576	0.99009901	0.048	Adjusted
Sakata 2019		Severe irAEs	0.25	0.0207053	3.018549	0.2866	Crude
Shankar 2020		irAEs	1.717402	1.049713	2.809786	0.0371	Crude
Sugano 2020		ILD	0.77	0.2	2.92	0.7	Adjusted
Suzuki 2020		ILD	0.840708	0.0930277	7.597627	1	Crude

Watanabe 2020		ILD	0.7985803	0.320584	1.989278	0.6285	Crude
Yamaguchi 2018		Pneumonitis	2.631579	0.4739337	50	0.31	Crude
Nakahama 2018	Pleural effusion	ILD	0.77	0.3	1.98	0.587	Adjusted
Owen 2018	PLR	irAEs	0.7058824	0.2858391	1.743183	0.4971	Crude
Aso 2020	Pre-existing anti-nuclear antibodies	skin reactions	1.96195	0.9737474	3.953025	0.0696	Crude
Chu 2020		Pneumonitis	0.902	0.329	2.474	0.841	Crude
Aso 2020	Pre-existing anti-thyroid antibodies	skin reactions	2.029825	0.8814161	4.674509	0.1196	Crude
Toi 2019	Pre-existing auto antibodies	irAEs	3.25	1.589025	6.647157	0.0017	Crude
Aso 2020	Pre-existing rheumatoid factor	skin reactions	3.41	1.58	7.36	0.002	Adjusted
Moey 2020	Previous atrial fibrillation/flutte	Inhibitor-Related Cardiotoxicity	2.390977	0.7138688	8.008157	0.2376	Crude
Moey 2020	Previous cardiovascular disease	Inhibitor-Related Cardiotoxicity	2.883951	1.113954	7.466349	0.0385	Crude
Suzuki 2020		ILD	0.281129653	0.01573833	5.0217459	0.39523904	Crude
Moey 2020	Previous cerebrovascular disease	Inhibitor-Related Cardiotoxicity	1.1475	0.3127615	4.210096	0.7379	Crude
Suzuki 2020		ILD	0.281129653	0.01573833	5.0217459	0.39523904	Crude
Moey 2020	Previous chronic kidney disease	Inhibitor-Related Cardiotoxicity	0.8282828	0.1000817	6.854923	1	Crude
Sugano 2020	Previous interstitial pneumonia	ILD	14.7	2.16	99.6	0.006	Adjusted
Nakahama 2018	Previous radiation pneumonitis	ILD	3.96	1.35	11.6	0.012	Adjusted
Nakanishi 2019		ILD	0.331	0.017	1.907	0.246	Crude
Nakahama 2018	Pulmonary metastasis	ILD	0.89	0.33	2.41	0.821	Adjusted
Fujita 2019	Radiotherapy before treatment	Infection diseases	1.200929	0.5307692	2.717247	0.6749	Crude
Fukihara 2019		Pneumonitis	0.888	0.362	2.178	0.795	Crude
Moey 2020		Inhibitor-Related Cardiotoxicity	1.005128	0.4179406	2.417288	1	Crude
Nakanishi 2019		ILD	0.622	0.158	2.069	0.449	Crude
Okada 2020		ILD	1.521739	0.5331625	4.34331	0.4192	Crude
Owen 2018		irAEs	0.926316	0.347419	2.469819	1	Crude
Sugano 2020		ILD	1.26	0.33	4.82	0.73	Adjusted
Nakanishi 2019	Reticular shadow	ILD	2.863	0.543	12.699	0.198	Crude
Fujita 2019	Rheumatoid arthritis	Infection diseases	1.056452	0.114054	9.785615	1	Crude
Aso 2020	Sex (Male vs female)	skin reactions	1.804444	0.7806822	4.170736	0.2328	Crude
Chu 2020		Pneumonitis	0.499	0.197	1.263	0.142	Adjusted
Cortellini 2020		irAEs	0.907	0.689	1.194	0.5257	Crude
Duma 2019		irAEs	0.4901961	0.2849003	0.8403361	0.008	Adjusted
Fujita 2019		Infection diseases	1.907468	0.7679182	4.738047	0.208121	Crude
Fukihara 2019		Pneumonitis	1.034	0.405	2.64	0.944	Crude
Kichenadasse 2020		irAEs	1.094691	0.8701623	1.377154	0.4492	Crude
Koyama 2019		immune-related thyroid dysfunction	0.71	0.25	2.05	0.532	Crude
Moey 2020		Inhibitor-Related Cardiotoxicity	1.401515	0.5644869	3.479699	0.5083	Crude
Nakahama 2018		ILD	1.98	0.55	7.07	0.296	Adjusted
Nakanishi 2019		ILD	1.094	0.324	4.344	0.889	Crude
Nishiyama 2019		Acute exacerbation of Interstitial lung disease	1.454545	0.1527713	13.84882	1	Crude
Okada 2020		ILD	3.258333	0.6971344	15.22911	0.1468	Crude

Sakata 2019		Severe irAEs	1.142857	0.2409135	5.421541	1	Crude
Shankar 2020		irAEs	1.062651	0.7550337	1.495597	0.7942	Crude
Sugano 2020		ILD	3.06	0.69	13.6	0.14	Adjusted
Suzuki 2020		ILD	5.107527	0.6517645	40.02493	0.1223	Crude
Watanabe 2020		ILD	1.188571	0.4853493	2.910691	0.8271	Crude
Yamaguchi 2018		Pneumonitis	1.724138	0.3355705	12.65823	0.53	Adjusted
Duma 2019		Severe irAEs	0.6486486	0.3031091	1.388098	0.3448	Crude
Zeng 2020	Simplified comorbidity score	irAEs	0.837606523	2.4990285	0.28074321	0.7899	Crude
Fukihara 2019	Smoking (pack-years)	Pneumonitis	0.994	0.981	1.007	0.362	Crude
Nishiyama 2019		Acute exacerbation of Interstitial lung disease	1.5	0.15	14.9	0.73	Adjusted
Okada 2020		ILD	3.01	1.04	8.72	0.042	Adjusted
Okada 2020		Severe irAEs	3.65	0.85	15.57	0.084	Adjusted
Aso 2020	Smoking status (Current/former vs Never)	Skin reactions	1.89759	0.7142208	5.041647	0.2607	Crude
Chu 2020		Pneumonitis	1.438	0.794	2.607	0.231	Crude
Fujita 2019		Infection diseases	2.038776	0.730449	5.69048	0.256161	Crude
Kichenadasse 2020		irAEs	1.120401	0.8303349	1.511799	0.4993	Crude
Koyama 2019		immune-related thyroid dysfunction	0.88	0.29	2.66	0.82	Crude
Nakahama 2018		ILD	1.24	0.27	5.69	0.778	Adjusted
Nakanishi 2019		ILD	3.925	0.696	73.997	0.136	Crude
Sakata 2019		Severe irAEs	1.477273	0.249775	8.737217	1	Crude
Shankar 2020		irAEs	1.097809	0.7031316	1.714025	0.7365	Crude
Sugano 2020		ILD	0.617283951	0.0862069	4.34782609	0.63	Adjusted
Suzuki 2020		ILD	1.727273	0.3698708	8.066252	0.7379	Crude
Watanabe 2020		ILD	1.893312	0.6299451	5.690386	0.3435	Crude
Yamaguchi 2018		Pneumonitis	2.63	0.69	17.35	0.17	Crude
Fujita 2019	Stage (xx vs. xx)	Infection diseases	1.263158	0.523248	3.049354	0.669521	Crude
Moey 2020		Inhibitor-Related Cardiotoxicity	0.4387464	0.1823601	1.055595	0.0669	Crude
Okada 2020		ILD	0.7212121	0.2279308	2.282039	0.5504	Crude
Sakata 2019		Severe irAEs	0.9625	0.229889	4.029793	1	Crude
Shankar 2020		irAEs	4.748858	2.004586	11.25003	0.0001	Crude
Suzuki 2020		ILD	1.083333	0.3279005	3.579168	1	Crude
Watanabe 2020		ILD	4.158416	0.5286081	32.71312	0.1985	Crude
Suresh 2018	Surgery	Pneumonitis	0.53	0.17	1.37	0.22	Crude
Koyama 2019	Thyroid dysfunction	Immune-related thyroid dysfunction	1.93	0.48	7.78	0.355	Crude
Owen 2018	TP53 mutation	irAEs	0.3966667	0.1472593	1.068486	0.101	Crude
Shankar 2020	Treatment combination	irAEs	0.1833333	0.0552085	0.608803	0.0011	Crude
Chu 2020		Pneumonitis	0.843	0.467	1.521	0.57	Crude
Suresh 2018	Chemotherapy	Pneumonitis	0.86	0.38	2	0.72	Crude
Chu 2020	Treatment line (1st vs $\geq 2^{nd}$)	Pneumonitis	0.783085356	0.41305246	1.4858841	0.455	Crude
Fukihara 2019		Pneumonitis	2.197	0.859	5.617	0.1	Crude
Kichenadasse 2020		irAEs	0.8669551	0.5895851	1.274814	0.505	Crude
Okada 2020		ILD	1.362637	0.4597171	4.038963	0.5743	Crude
Sakata 2019		Severe irAEs	1.75	0.4807723	6.369959	0.5113	Crude
Suzuki 2020		ILD	6.157895	0.3692995	102.68	0.2698	Crude
Watanabe 2020		ILD	1.197133	0.4565096	3.139313	0.7974	Crude

Yamaguchi 2018		Pneumonitis	1.29	0.34	4.09	0.68	Crude
Koyama 2019	Treatment line (≥3rd line)	immune-related thyroid dysfunction	1.58	0.59	4.21	0.359	Crude
Shankar 2020	Treatment response	irAEs	3.806452	2.60527	5.561449	0	Crude
Suzuki 2020		ILD	2.201058	0.8197924	5.909615	0.1474	Crude
Koyama 2019	TTF1 expression	immune-related thyroid dysfunction	0.37	0.13	1.05	0.062	Adjusted
Sakata 2019	Tumor burden	Severe irAEs	8.625	1.960687	37.941093	0.004592	Crude
Moey 2020	Type of lung tumor (NSCLC)	Inhibitor-Related Cardiotoxicity	0.664557	0.177431	2.489058	0.4653	Crude
Nishiyama 2019	UIP radiological	Acute exacerbation of Interstitial lung disease	1.32	0.07	25.5	0.85	Adjusted
Okada 2020	Use of EGFR-TKI	ILD	0.452991453	0.02339197	8.77229421	0.61285302	Crude
Moey 2020	Use of alkylating agents	Inhibitor-Related Cardiotoxicity	2.568345	0.5741704	11.48857	0.2614	Crude
Moey 2020	Use of anti-topoisomerase drugs	Inhibitor-Related Cardiotoxicity	1.042208	0.4272149	2.542507	1	Crude
Moey 2020	Use of anti-VEGF drugs	Inhibitor-Related Cardiotoxicity	0.252955083	0.01454847	4.39814372	0.35110632	Crude
Moey 2020	Use of antimetabolites	Inhibitor-Related Cardiotoxicity	1.415441	0.5911256	3.389252	0.4998	Crude
Moey 2020	Use of beta-blocker	Inhibitor-Related Cardiotoxicity	5.467787	2.122235	14.08736	0.0003	Crude
Moey 2020	Use of calcium channel blocker	Inhibitor-Related Cardiotoxicity	0.8606811	0.2748322	2.695361	1	Crude
Moey 2020	Use of loop diuretics	Inhibitor-Related Cardiotoxicity	3.860317	1.460686	10.20209	0.009	Crude
Moey 2020	Use of renin-angiotensin-aldosterone system inhibitors	Inhibitor-Related Cardiotoxicity	0.8943089	0.3126544	2.55806	1	Crude
Moey 2020	Use of statins	Inhibitor-Related Cardiotoxicity	2.163009	0.8999508	5.198739	0.1043	Crude
Chu 2020	Use of steroids	Pneumonitis	0.729	0.362	1.467	0.375	Crude
Fujita 2019		Infection diseases	1.905594	0.805369	4.508851	0.15443	Crude
Moey 2020		Inhibitor-Related Cardiotoxicity	6.609836	2.339224	18.6771	0.0002	Crude
Moey 2020	Use of taxanes	Inhibitor-Related Cardiotoxicity	5.197861	1.70758	15.82225	0.007	Crude
Moey 2020	Use of thiazide diuretic	Inhibitor-Related Cardiotoxicity	1.1475	0.3127615	4.210096	0.7379	Crude
Fukihara 2019	White Blood Cell count	Pneumonitis	1.09	1	1.188	0.051	Crude
Nakanishi 2019		ILD	1.00005	0.99999	1.0002	0.062	Crude

PD-L1= Programmed death-ligand 1, NSCLC= Non-small-cell lung carcinoma, irAEs= Immune related adverse events, ALK= Anaplastic lymphoma kinase, COPD= Chronic obstructive pulmonary disease, EGFR= epidermal growth factor receptor, ILD= Interstitial lung disease, CRP= *C-reactive protein,* CT= Computed tomography, ALT= Alanine transaminase, AST= Aspartate transaminase, NLR= neutrophil to lymphocyte ratio, RT= Radiotherapy, TTF1= Transcription Termination Factor 1, LDH= Lactate dehydrogenase, ICI= Immune check-point inhibitors, UIP= Usual interstitial pneumonia, CNS= central nervous system, PLR= Platelet lymphocyte ratio, IAOT= imaging findings of airway obstruction adjacent to lung tumors, CRP= C-reactive protein, EGFR-TKI = Epidermal growth factor receptor tyrosine kinase inhibitors.

Supplementary Table 2. Random effects meta-analysis. Pooled adjusted odds ratios reflecting the association between risk factors and irAEs in patients treated with ICIs.

Risk Factor	Pooled OR	LCI	UCI	Chi-2	Chi-2 p value	I2	P value	Number of Studies included	Number of participants
Age (≥65 vs <65)	1.054	0.642	1.73	6.9	0.141	42.00%	0.837	5[1][2][3][4][5]	777
**Ground glass attenuation (Ground glass attenuation vs normal CT)**	**77.098**	**7.818**	**760.304**	**0.88**	**0.349**	**0.00%**	**0.0001**	**2[4][6]**	**131**
**Histology (Squamous vs non-squamous)**	**1.847**	**1.048**	**3.256**	**1.62**	**0.445**	**0.00%**	**0.034**	**3[7][5][8]**	**536**
Performance status (≥2 vs 0-1)	0.697	0.334	1.453	3.62	0.164	44.8%	0.335	3[4][5][9]	433
**Smoking pack-years (≥50 vs <50 packs/year)**	**2.662**	**1.014**	**6.989**	**0.29**	**0.59**	**0.00%**	**0.047**	**2[4][9]**	**331**
Smoking status (Current/former vs never smoker)	0.953	0.286	3.176	0.3	0.582	0.00%	0.938	2[7][5]	331
Sex (Male vs female)	0.696	0.462	1.049	9.43	0.051	57.60%	0.083	5[3][7][5][10][11]	885

OR= Odds Ratio, LCI= Lower Confidence Interval, UCI= Upper confidence interval

Supplementary Table 3. Stratified analysis, random effects meta-analysis by type of irAE. Pooled crude and adjusted odds ratios reflecting the association between risk factors and irAEs in patients treated with ICIs.
.

Risk Factor	Type of irAE	Pooled OR	LCI	UCI	X2	X2 p value	I2	P value	Number of studies includes	Total number of participants
Age<br>(≥65 vs <65)	Pneumonitis	1.546	0.888	2.692	0.36	0.549	0.0%	0.123	3[3][10][8]	625
Age<br>(≥65 vs <65)	irAEs	1.122	0.875	1.439	3.05	0.081	67.20%	0.363	2[12][13]	1295
COPD<br>(History of COPD vs no history of COPD)	Interstitial lung disease	1.166	0.625	2.175	4.31	0.116	53.60%	0.629	3[6][9][14]	323
Histology<br>(Squamous vs non-squamous)	Pneumonitis	1.276	0.799	38	2.05	0.36	2.2%%	0.308	3[3][10][15]	593
Histology<br>(Squamous vs non-squamous)	Interstitial lung disease	1.388	0.89	2.163	9.01	0.109	44.50%	0.148	6[6][7][5][9][14][16]	885
Histology<br>(Squamous vs non-squamous)	irAEs	0.827	0.693	0.985	0.29	0.867	0.00%	0.033	3[13][17][18]	3181
Pembrolizumab treatment<br>(Pembrolizumab treatment vs other ICIs)	Pneumonitis	1.841	0.866	3.913	6.46	0.011	84.50%	0.113	2[15][8]	375
LDH<br>(≥240 vs <240 U/L)	Interstitial lung disease	1	0.999	1.001	2.36	0.124	57.60%	0.754	2[6][7]	284
Performance status<br>(≥2 vs 0-1)	Pneumonitis	0.973	0.441	2.146	3.21	0.201	37.6%	0.946	3[3][10][15]	593
Performance status<br>(≥2 vs 0-1)	Interstitial lung disease	1.309	0.773	2.217	3.80	0.578	0.0%	0.316	6[6][7][5][9][14][16]	883
**Performance status<br>(≥2 vs 0-1)**	**Severe irAEs**	**4.274**	**1.215**	**14.925**	**0.00**	**0.953**	**0.0%**	**0.024**	**2[9][19]**	**144**
**Performance status<br>(≥2 vs 0-1)**	**irAEs**	**0.598**	**0.449**	**0.797**	**3.84**	**0.279**	**21.8%**	**0.0001**	**4[12] [13][17][18]**	**3466**
PD-L1 expression<br>(≥50% vs. <50%)	Interstitial lung disease	1.898	0.92	3.913	0.17	0.918	0.00%	0.083	3[9][14][16]	471
Previous radiation pneumonitis<br>(History of radiation pneumonitis vs<br>no history of radiation pneumonitis)	Interstitial lung disease	2.584	0.971	6.877	3.52	0.061	71.60%	0.057	2[6][7]	284
Radiotherapy<br>(Previous radiotherapy vs no radiotherapy)	Interstitial lung disease	1.114	0.556	2.232	1.16	0.56	0.00%	0.761	3[6][5][9]	315
Smoking status<br>(Current/former vs never smoker)	Pneumonitis	1.546	0.885	2.7	0.47	0.491	0.00%	0.126	2[3][10]	423
Smoking status<br>(Current/former vs never smoker)	Interstitial lung disease	1.584	0.797	3.147	1.68	0.794	0.00%	0.189	5[6][7][5][14][16]	783
Smoking status<br>(Current/former vs never smoker)	irAEs	1.113	0.868	1.428	0.01	0.941	0.00%	0.397	2[17][18]	2171
Sex<br>(Male vs female)	Pneumonitis	0.793	0.427	1.475	1.97	0.374	0.00%	0.464	3[3][10][15]	593
**Sex<br>(Male vs female)**	**Interstitial lung disease**	**1.79**	**1.054**	**3.041**	**3.45**	**0.631**	**0.00%**	**0.031**	**6[6][7][5][9][14][16]**	**885**

Sex<br>(Male vs female)	irAEs	0.967	0.832	1.124	7.68	0.053	61.00%	0.66	4[11][13][17][18]	3412
Sex<br>(Male vs female)	Severe irAEs	0.723	0.365	1.433	0.41	0.522	0.00%	0.353	2[11][19]	273
**Sex<br>(Male vs female)**	**Skin reactions**	**2.351**	**1.159**	**4.769**	**1.33**	**0.248**	**24.9%**	**0.018**	**2[11][20]**	**386**
Stage<br>(Stage III/IV vs I/II)	Interstitial lung disease	1.089	0.505	2.352	2.11	0.348	5.30%	0.827	3[9][16][14]	471
Treatment line<br>(First vs second/third line)	Pneumonitis	1.115	0.686	1.815	3.23	0.199	38.00%	0.66	3[3][10][15]	590
Treatment line<br>(First vs second/third line)	Interstitial lung disease	1.397	0.695	2.809	1.17	0.558	0.00%	0.348	3[9][14][16]	471

OR= Odds Ratio, LCI= Lower Confidence Interval, UCI= Upper confidence interval, COPD=Chronic Obstructive Pulmonary Disease, PD-L1=Programmed Death-ligand 1, LDH= Lactate dehydrogenase, U/L=Units per liter, mg/dL= milligrams per deciliter.

Supplementary Table 4. Stratified analysis, random effects meta-analysis by quality/risk of bias. Pooled crude and adjusted odds ratios reflecting the association between risk factors and irAEs in patients treated with ICIs.

Risk Factor	Quality/risk of bias	Pooled OR	LCI	UCI	Chi-2	Chi-2 p value	I2	P value	Number of studies includes	Total number of participants
LDH<br>(≥240 vs <240 U/L)	Moderate quality/Moderate risk of bias	1	0.999	1.001	4.04	0.132	50.50%	0.753	3[6][7][10]	407
Pembrolizumab treatment<br>(Pembrolizumab treatment vs other ICIs)	High quality/Low risk of bias	1.841	0.866	3.913	6.46	0.011	84.50%	0.113	2[8] [15]	375
Previous radiation pneumonitis<br>(History of radiation pneumonitis vs no history of radiation pneumonitis)	Moderate quality/Moderate risk of bias	2.584	0.971	6.877	3.52	0.061	71.60%	0.057	2[6][7]	284
Stage<br>(Stage III/IV vs I/II)	Low quality/High risk of bias	1.38	0.784	2.428	14.67	0.001	86.40%	0.265	3[21][19][18]	861
Stage<br>(Stage III/IV vs I/II)	Moderate quality/Moderate risk of bias	1.365	0.698	2.67	1.29	0.524	0.00%	0.363	3[14][2][16]	536
Combination treatment<br>(ICI + chemotherapy vs monotherapy)	High quality/Low risk of bias	0.849	0.525	1.373	0	0.969	0.00%	0.504	2[8][3]	505

OR= Odds Ratio, LCI= Lower Confidence Interval, UCI= Upper confidence interval, COPD=Chronic Obstructive Pulmonary Disease, ILD= Interstitial lung disease, PD-L1=Programmed Death-ligand 1, LDH= Lactate dehydrogenase, U/L=Units per liter, mg/dL= milligrams per deciliter.

Supplementary Table 5. Stratified analysis, random effects meta-analysis by region. Pooled odds ratios of Crude and adjusted odds ratios reflecting the association between risk factors and irAEs in patients treated with ICIs.

Risk Factor	Region	Pooled OR	LCI	UCI	Chi-2	Chi-2 p value	I2	P value	Number of studies includes	Total number of participants
LDH (≥240 vs <240 U/L)	Asia	1	0.999	1.001	4.04	0.132	50.50%	0.753	3[7][6][10]	407
**Pembrolizumab treatment (Pembrolizumab treatment vs other ICIs)**	**Asia**	**1.973**	**1.12**	**3.476**	**3.88**	**0.143**	**48.50%**	**0.019**	**3[15][19][16]**	**443**
Previous radiation pneumonitis (History of radiation pneumonitis vs no history of radiation pneumonitis)	Asia	2.584	0.971	6.877	3.52	0.061	71.60%	0.057	2[7][6]	284
Stage (Stage III/IV vs I/II)	USA	1.474	0.797	2.728	14.39	0	93.00%	0.216	2[21][18]	819
Stage (Stage III/IV vs I/II)	Asia	1.131	0.661	1.936	2.23	0.693	0.00%	0.653	5[2][9][19][14][16]	680
Combination treatment (ICI + chemotherapy vs monotherapy)	USA	0.521	0.263	1.032	4.31	0.038	76.80%	0.062	2[8][18]	828
Use of steroids (Previous use of steroids vs no previous use of steroids)	Asia	1.068	0.621	1.839	2.88	0.09	65.30%	0.812	2[2][3]	467
White Blood Cell count (≥10^3 vs <10^3 /mm^3)	Asia	1	1	1	3.84	0.05	74.00%	0.35	2[15][6]	253

OR= Odds Ratio, LCI= Lower Confidence Interval, UCI= Upper confidence interval, CRP= C-Reactive protein

Supplementary Table 6. Risk factors for developing common irAEs. Crude and adjusted odds ratios reflecting the association between risk factors and irAEs in patients treated with ICIs.

A**uthor**/Year	**Risk factor**	**Type of irAE**	**OR**	**LCI**	**UCI**	**P value**	**Type of analysis**
Aso 2020	Preexisting anti-nuclear antibodies (Preexisting anti-nuclear antibodies vs no preexisting anti-nuclear antibodies)	Skin reactions	1.96195	0.9737474	3.953025	0.0696	Crude
Aso 2020	Preexisting anti-thyroid antibodies (Preexisting anti-thyroid antibodies vs no preexisting anti-thyroid antibodies)	Skin reactions	2.029825	0.8814161	4.674509	0.1196	Crude
Metro 2020	Brain metastasis (Brain metastasis vs no brain metastasis)	Acute respiratory distress syndrome	0.43756146	0.02321661	8.24668435	0.59321383	Crude
Metro 2020	Brain metastasis (Brain metastasis vs no brain metastasis)	Adrenal insufficiency	1.33038348	0.05347961	33.0952368	0.87159408	Crude
Metro 2020	Brain metastasis (Brain metastasis vs no brain metastasis)	Colitis	2.055556	0.1809565	14.72148	0.3411	Crude
Metro 2020	Brain metastasis (Brain metastasis vs no brain metastasis)	Hepatitis	8.222222	0.4164521	487.1336	0.1036	Crude
Metro 2020	Brain metastasis (Brain metastasis vs no brain metastasis)	Mucositis	11.9734513	0.48131645	297.857132	0.13013106	Crude
Metro 2020	Brain metastasis (Brain metastasis vs no brain metastasis)	Myocarditis	1.33038348	0.05347961	33.0952368	0.87159408	Crude
Metro 2020	Brain metastasis (Brain metastasis vs no brain metastasis)	Myositis	0.79469027	0.0376206	16.7868846	0.89141954	Crude
Metro 2020	Brain metastasis (Brain metastasis vs no brain metastasis)	Peripheral neuropathy	1.33038348	0.05347961	33.0952368	0.87159408	Crude
Metro 2020	Brain metastasis (Brain metastasis vs no brain metastasis)	Skin rash	1.33038348	0.05347961	33.0952368	0.87159408	Crude
Metro 2020	Brain metastasis (Brain metastasis vs no brain metastasis)	Toxic epidermal necrolysis	1.33038348	0.05347961	33.0952368	0.87159408	Crude
Zeng 2020	CCI (<1 vs ≥1)	Colitis	0.35526316	0.00934471	6.06646672	0.39187828	Crude
Zeng 2020	CCI (<1 vs ≥1)	Hepatitis	0.3513514	0.0058017	7.192269	0.5698	Crude
Zeng 2020	CCI (<1 vs ≥1)	Hypothyroidism	0.2252252	0.0041798	3.053743	0.3036	Crude
Zeng 2020	CCI (<1 vs ≥1)	Pneumonitis	0.7142857	0.0886452	5.811392	0.6929	Crude
Zeng 2020	CCI (<1 vs ≥1)	Skin rash	4.090909	0.4136998	200.4955	0.2303	Crude
Aso 2020	ECOG Performance status (>=2 vs 0-1)	Skin reactions	0.4803922	0.06571	3.512045	0.5985	Crude
Aso 2020	Histology	Skin reactions	1.121528	0.5587607	2.251097	0.8584	Crude
Aso 2020	PD-L1 (≥1% vs <1%)	Skin reactions	2.3	0.696761	7.592269	0.191796	Crude
**Aso 2020**	**Preexisting rheumatoid factor**	**Skin reactions**	**3.41**	**1.58**	**7.36**	**0.002**	**Adjusted**
Zeng 2020	SCS (<8 vs ≥8)	Colitis	1.25	0.07406568	48.1721239	0.71349524	Crude
Zeng 2020	SCS (<8 vs ≥8)	Hepatitis	1.230769	0.0607903	75.58945	1	Crude
Zeng 2020	SCS (<8 vs ≥8)	Hypothyroidism	0.5897436	0.0405429	8.718642	0.6302	Crude

Zeng 2020	SCS (<8 vs ≥8)	Pneumonitis	3.333333	0.3366566	164.0923	0.3958	Crude
Zeng 2020	SCS (<8 vs ≥8)	Skin reactions	0.5789474	0.0718143	4.74271	0.6656	Crude
Duma 2019	Sex (Male vs female)	Arthralgia	0.2090909	0.0043817	1.921812	0.2148	Crude
Duma 2019	Sex (Male vs female)	Colitis	1.084112	0.1967044	5.97113	1	Crude
**Duma 2019**	**Sex (Male vs female)**	**Endocrinopathies**	**0.1154062**	**0.0127169**	**0.5094497**	**0.0006**	**Crude**
Duma 2019	Sex (Male vs female)	Hepatitis	0.4484127	0.0732122	2.033137	0.3367	Crude
**Duma 2019**	**Sex (Male vs female)**	**Skin reactions**	**4.53125**	**1.374495**	**19.26319**	**0.0071**	**Crude**
Aso 2020	Sex (Male vs female)	Skin reactions	1.804444	0.7806822	4.170736	0.2328	Crude
Aso 2020	Smoking (Ever vs never)	Skin reactions	1.89759	0.7142208	5.041647	0.2607	Crude

OR= Odds Ratio, LCI= Lower Confidence Interval, UCI= Upper confidence interval, CCI= Charlson comorbidity index, SCS= Simplified comorbidity score

References

[1] Koyama J, Horiike A, Yoshizawa T, Dotsu Y, Ariyasu R, Saiki M, et al. Correlation between thyroid transcription factor-1 expression, immune-related thyroid dysfunction, and efficacy of anti-programmed cell death protein-1 treatment in non-small cell lung cancer. J Thorac Dis 2019;11:1919–28. https://doi.org/10.21037/jtd.2019.04.102.

[2] Fujita K, Kim YH, Kanai O, Yoshida H, Mio T, Hirai T. Emerging concerns of infectious diseases in lung cancer patients receiving immune checkpoint inhibitor therapy. Respir Med 2019;146:66–70. https://doi.org/10.1016/j.rmed.2018.11.021.

[3] Chu X, Zhao J, Zhou J, Zhou F, Jiang T, Jiang S, et al. Association of baseline peripheral-blood eosinophil count with immune checkpoint inhibitor-related pneumonitis and clinical outcomes in patients with non-small cell lung cancer receiving immune checkpoint inhibitors. Lung Cancer 2020;150:76–82. https://doi.org/10.1016/j.lungcan.2020.08.015.

[4] Nishiyama N, Honda T, Sema M, Kawahara T, Jin Y, Natsume I, et al. The utility of ground-glass attenuation score for anticancer treatment-related acute exacerbation of interstitial lung disease among lung cancer patients with interstitial lung disease. Int J Clin Oncol 2020;25:282–91. https://doi.org/10.1007/s10147-019-01576-x.

[5] Sugano T, Seike M, Saito Y, Kashiwada T, Terasaki Y, Takano N, et al. Immune checkpoint inhibitor-associated interstitial lung diseases correlate with better prognosis in patients with advanced non-small-cell lung cancer. Thorac Cancer 2020;11:1052–60. https://doi.org/10.1111/1759-7714.13364.

[6] Nakanishi Y, Masuda T, Yamaguchi K, Sakamoto S, Horimasu Y, Nakashima T, et al. Pre-existing interstitial lung abnormalities are risk factors for immune checkpoint inhibitor-induced interstitial lung disease in non-small cell lung cancer. Respir Investig 2019;57:451–9. https://doi.org/10.1016/j.resinv.2019.05.002.

[7] Nakahama K, Tamiya A, Isa S-I, Taniguchi Y, Shiroyama T, Suzuki H, et al. Association Between Imaging Findings of Airway Obstruction Adjacent to Lung Tumors and the Onset of Interstitial Lung Disease After Nivolumab. In Vivo 2018;32:887–91. https://doi.org/10.21873/invivo.11324.

[8] Suresh K, Voong KR, Shankar B, Forde PM, Ettinger DS, Marrone KA, et al. Pneumonitis in Non-Small Cell Lung Cancer Patients Receiving Immune Checkpoint Immunotherapy: Incidence and Risk Factors. J Thorac Oncol 2018;13:1930—1939. https://doi.org/10.1016/j.jtho.2018.08.2035.

[9] Okada N, Matsuoka R, Sakurada T, Goda M, Chuma M, Yagi K, et al. Risk factors of immune checkpoint inhibitor-related interstitial lung disease in patients with lung cancer: a single-institution retrospective study. Sci Rep 2020;10:1–9. https://doi.org/10.1038/s41598-020-70743-2.

[10] Yamaguchi T, Shimizu J, Hasegawa T, Horio Y, Inaba Y, Yatabe Y, et al. Pre-existing pulmonary fibrosis is a risk factor for anti-PD-1-related pneumonitis in patients with non-small cell lung cancer: A retrospective analysis. Lung Cancer 2018;125:212–7. https://doi.org/10.1016/j.lungcan.2018.10.001.

[11] Duma N, Abdel-Ghani A, Yadav S, Hoversten KP, Reed CT, Sitek AN, et al. Sex Differences in Tolerability to Anti-

Programmed Cell Death Protein 1 Therapy in Patients with Metastatic Melanoma and Non-Small Cell Lung Cancer: Are We All Equal? Oncologist 2019;24:e1148–55. https://doi.org/10.1634/theoncologist.2019-0094.
[12] Ahmed T, Lycan T, Dothard A, Ehrlichman P, Ruiz J, Farris M, et al. Performance Status and Age as Predictors of Immunotherapy Outcomes in Advanced Non–Small-Cell Lung Cancer. Clin Lung Cancer 2020;21:e286–93. https://doi.org/10.1016/j.cllc.2020.01.001.
[13] Cortellini A, Friedlaender A, Banna GL, Porzio G, Bersanelli M, Cappuzzo F, et al. Immune-related Adverse Events of Pembrolizumab in a Large Real-world Cohort of Patients With NSCLC With a PD-L1 Expression ≥ 50% and Their Relationship With Clinical Outcomes. Clin Lung Cancer 2020;21:498-508.e2. https://doi.org/10.1016/j.cllc.2020.06.010.
[14] Suzuki Y, Karayama M, Uto T, Fujii M, Matsui T, Asada K, et al. Assessment of Immune-Related Interstitial Lung Disease in Patients With NSCLC Treated with Immune Checkpoint Inhibitors: A Multicenter Prospective Study. J Thorac Oncol 2020;15:1317–27. https://doi.org/10.1016/j.jtho.2020.04.002.
[15] Fukihara J, Sakamoto K, Koyama J, Ito T, Iwano S, Morise M, et al. Prognostic Impact and Risk Factors of Immune-Related Pneumonitis in Patients With Non-Small-Cell Lung Cancer Who Received Programmed Death 1 Inhibitors. Clin Lung Cancer 2019;20:442-450.e4. https://doi.org/10.1016/j.cllc.2019.07.006.
[16] Watanabe S, Ota T, Hayashi M, Ishikawa H, Otsubo A, Shoji S, et al. Prognostic significance of the radiologic features of pneumonitis induced by anti-PD-1 therapy. Cancer Med 2020;9:3070–7. https://doi.org/10.1002/cam4.2974.
[17] Kichenadasse G, Miners JO, Mangoni AA, Rowland A, Hopkins AM, Sorich MJ. Multiorgan immune-related adverse events during treatment with atezolizumab. JNCCN J Natl Compr Cancer Netw 2020;18:1191–9. https://doi.org/10.6004/jnccn.2020.7567.
[18] Shankar B, Zhang J, Naqash AR, Forde PM, Feliciano JL, Marrone KA, et al. Multisystem Immune-Related Adverse Events Associated with Immune Checkpoint Inhibitors for Treatment of Non-Small Cell Lung Cancer. JAMA Oncol 2020:1–6. https://doi.org/10.1001/jamaoncol.2020.5012.
[19] Sakata Y, Kawamura K, Ichikado K, Shingu N, Yasuda Y, Eguchi Y, et al. The association between tumor burden and severe immune-related adverse events in non-small cell lung cancer patients responding to immune-checkpoint inhibitor treatment. Lung Cancer 2019;130:159–61. https://doi.org/10.1016/j.lungcan.2019.02.011.
[20] Aso M, Toi Y, Sugisaka J, Aiba T, Kawana S, Saito R, et al. Association Between Skin reactions and Clinical Benefit in Patients Treated with Anti-Programmed Cell Death 1 Monotherapy for Advanced Non-Small Cell Lung Cancer. Oncologist 2020;25:e536–44. https://doi.org/10.1634/theoncologist.2019-0550.
[21] Moey MYY, Tomdio AN, McCallen JD, Vaughan LM, O'Brien K, Naqash AR, et al. Characterization of Immune Checkpoint Inhibitor-Related Cardiotoxicity in Lung Cancer Patients From a Rural Setting. JACC CardioOncology 2020;2:491–502. https://doi.org/10.1016/j.jaccao.2020.07.005.
